# Supplementary material for: Alkaloids with Anti-Onchocercal Activity from Voacanga africana Stapf (Apocynaceae): Identification and Molecular Modeling
Source: Molecules. 2020 Dec 25;26(1):70. doi: 10.3390/molecules26010070 (PMC7795662; doi:10.3390/molecules26010070)
Supplement: Supplementary file 1 [file molecules-26-00070-s001.pdf]

SUPPLEMENTARY INFORMATION

Figure S1: IR spectrum of voacamine A.

Figure S2: Mass spectrum of voacamine A.

Figure S3: ^{1}H NMR (500 MHz, $CDCl_3$) spectrum of voacamine A.

Figure S4: ^{13}C NMR (125 MHz, $CDCl_3$) spectrum of voacamine A.

Figure S5: ^{1}H-^{1}H COSY spectrum of voacamine A.

Figure S6: ^{1}H-^{1}H NOESY spectrum of voacamine A.

Figure S7: ^{1}H-^{1}H HSQC spectrum of voacamine A.

Figure S8: ^{1}H-^{1}H HSQC and ^{1}H-^{13}C HMBC spectra of voacamine A.

Figure S9: ^{1}H-^{15}N-HSQC and ^{1}H-^{15}N-HMBC spectra of voacamine A.

Figure S10: ^{1}H NMR (500 MHz, $CDCl_3$) spectrum of voacangine.

Figure S11: ^{13}C NMR (125 MHz, $CDCl_3$) spectrum of voacangine.

Figure S12: ^{1}H NMR (500 MHz, $CDCl_3$) spectrum of voacristine.

Figure S13: ^{13}C NMR (125 MHz, $CDCl_3$) spectrum of voacristine.

Figure S14: ^{1}H NMR (500 MHz, $CDCl_3$) spectrum of coronaridine.

Figure S15: ^{13}C NMR (125 MHz, $CDCl_3$) spectrum of coronaridine.

Figure S16: ^{1}H NMR (500 MHz, $CDCl_3$) spectrum of tabernanthine.

Figure S17: ^{13}C NMR (125 MHz, DMSO-d_6)) spectrum of tabernanthine.

Figure S18: ^{1}H NMR (500 MHz,MeOD) spectrum of iboxygaine.

Figure S19: ^{13}C NMR (125 MHz, $CDCl_3$) spectrum of iboxygaine.

Figure S20: ^{1}H NMR (500 MHz, DMSO-d_6) spectrum of voacamine.

Figure S21: ^{13}C NMR (125 MHz, DMSO-d_6) spectrum of voacamine.

Figure S22: ^{1}H NMR (500 MHz, $CDCl_3$) spectrum of voacorine.

Figure S23: ^{13}C NMR (125 MHz, $CDCl_3$) spectrum of voacorine.

Figure S24: ^{1}H NMR (500 MHz, $CDCl_3$) spectrum of conoduramine.

Figure S25: ^{13}C NMR (125 MHz, $CDCl_3$) spectrum of conoduramine.

Figure S26:Percentage sequence identity and similarity values to our target.

Figure S27:Ramachandran plot (φ/ψ) distribution of the backbone conformation.

Figure S28: Chemical structure of auranofin.

Figure S29: Docking poses of (A) compound **1a**, (B) compound **5**, (C) compound **6**, (D) compound **7a**.

Figure S30: Docking poses of (A) compound **7**, (B) compound **8**, (C) compound **9**, (D) compound **9b**.

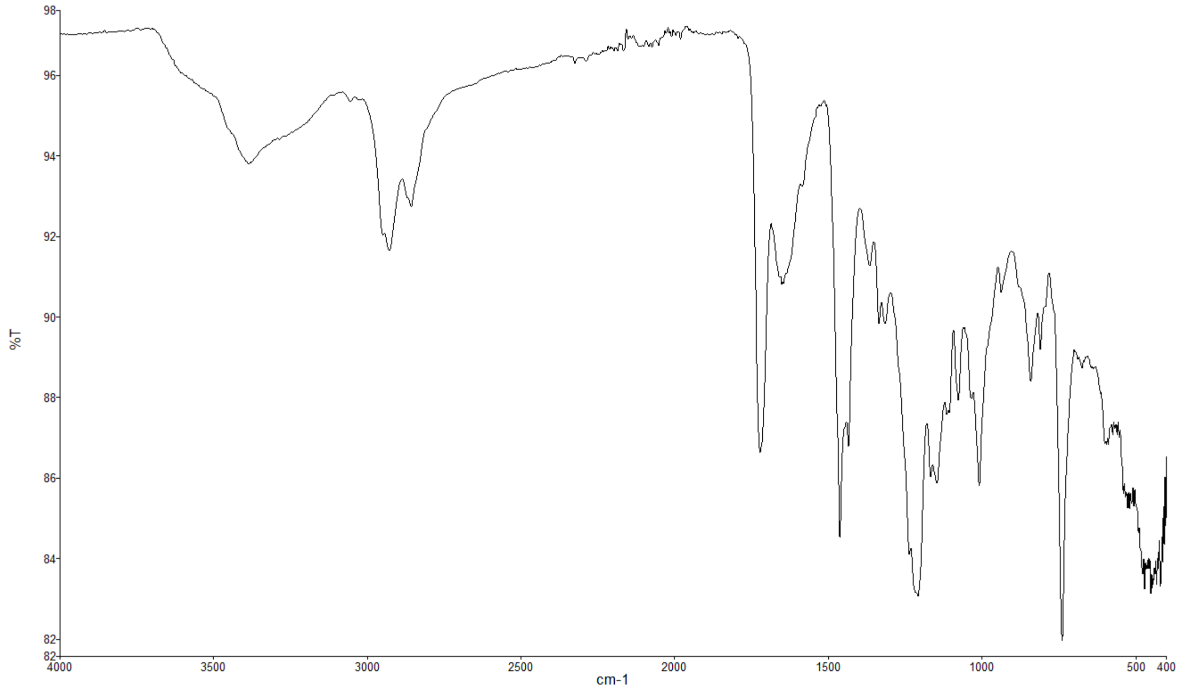

Figure S1: IR spectrum of voacamine A.

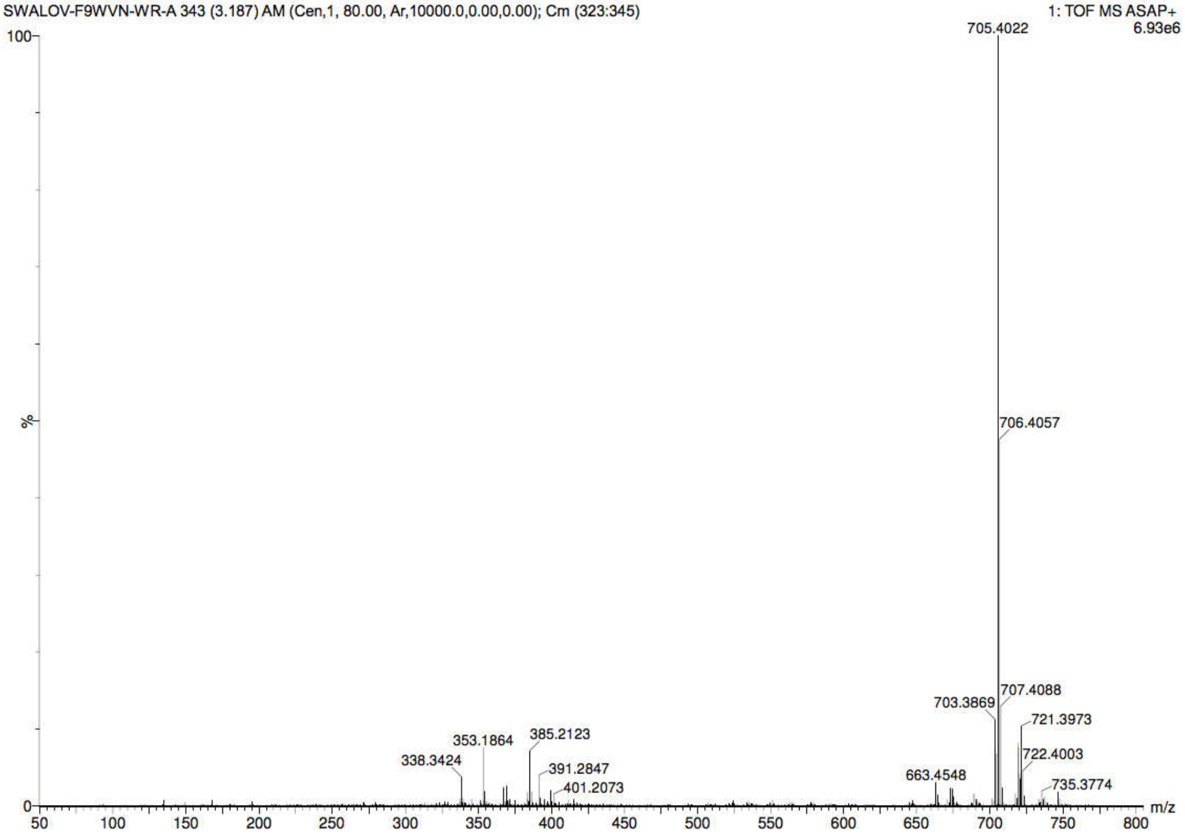

Figure S2: Positive-ion mass spectrum of voacamine A.

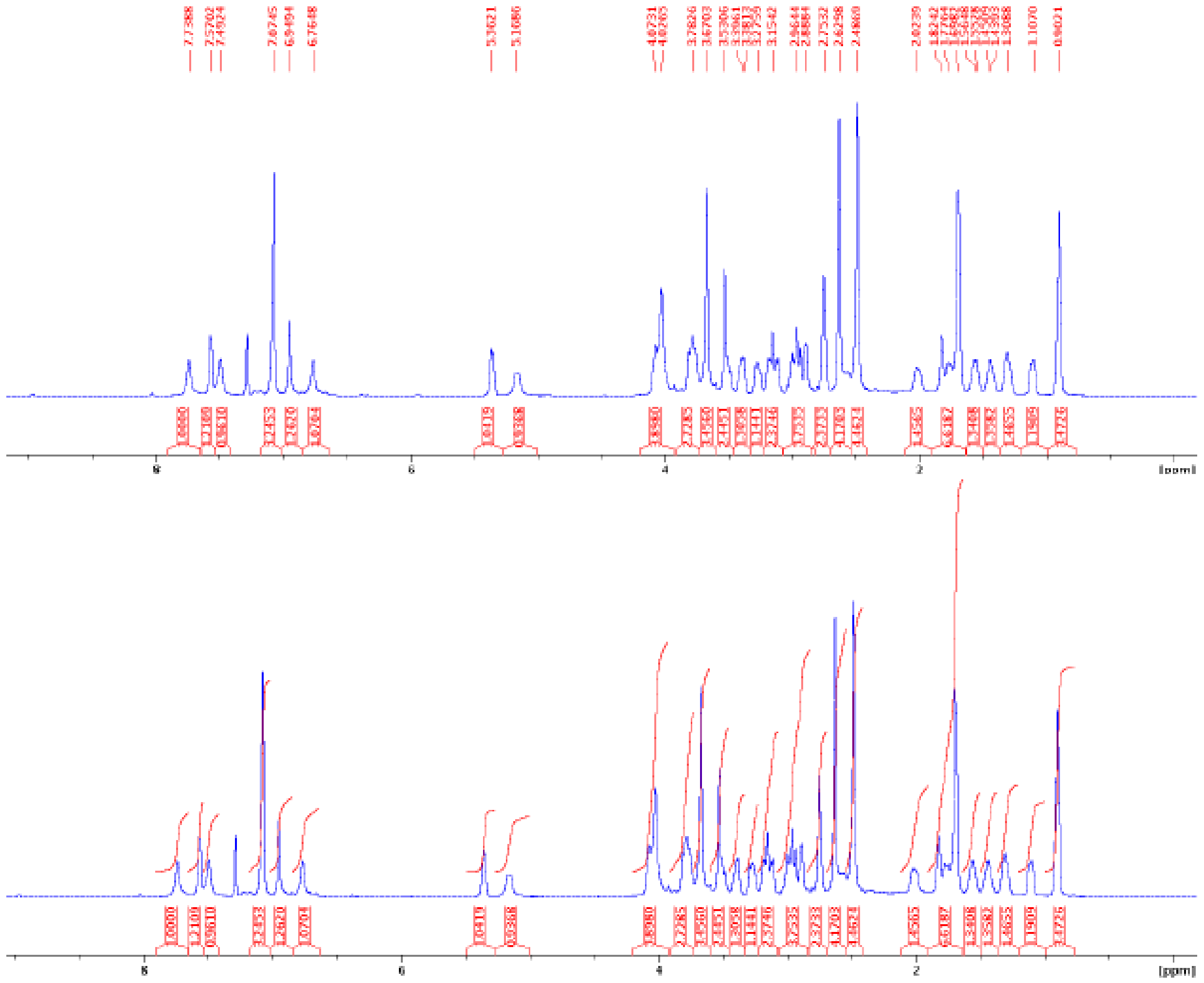

Figure S3: ^{1}H NMR (500 MHz, $CDCl_3$) spectrum of voacamine A.

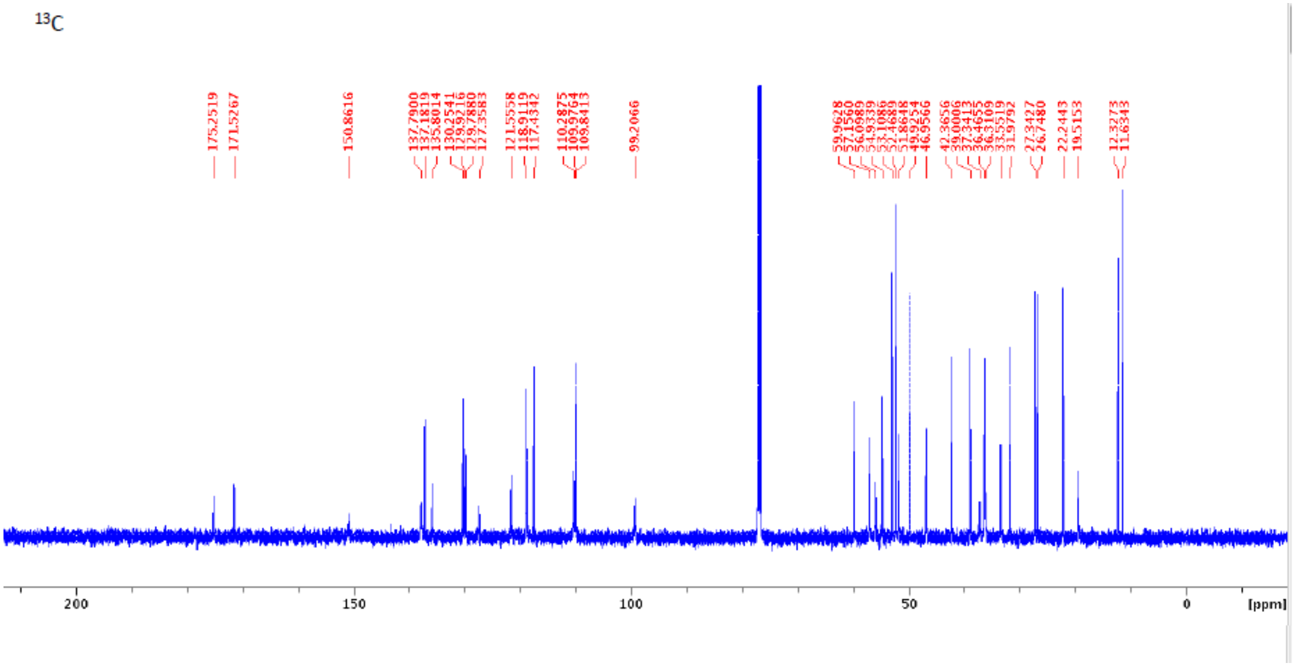

Figure S4: ^{13}C NMR (125 MHz, $CDCl_3$) spectrum of voacamine A.

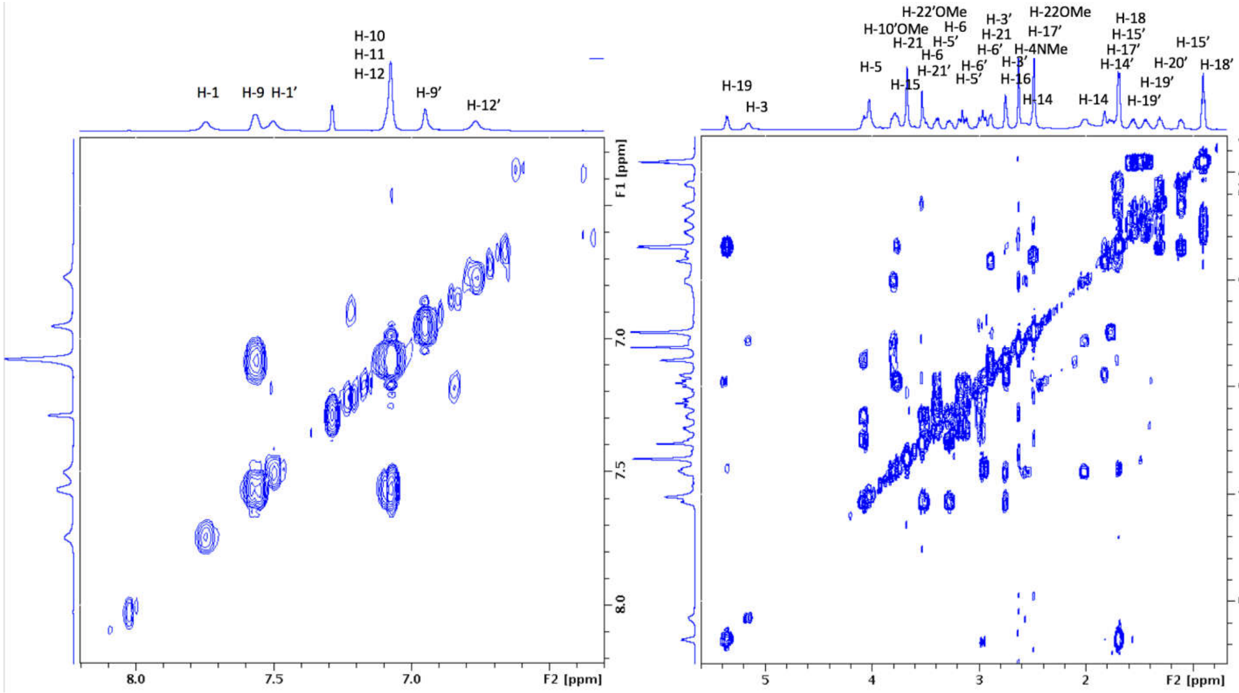

Figure S5: ^{1}H-^{1}H COSY spectrum of voacamine A. Atom labels on the 1D spectrum are positioned so the centre of the H corresponds to the resonant frequency.

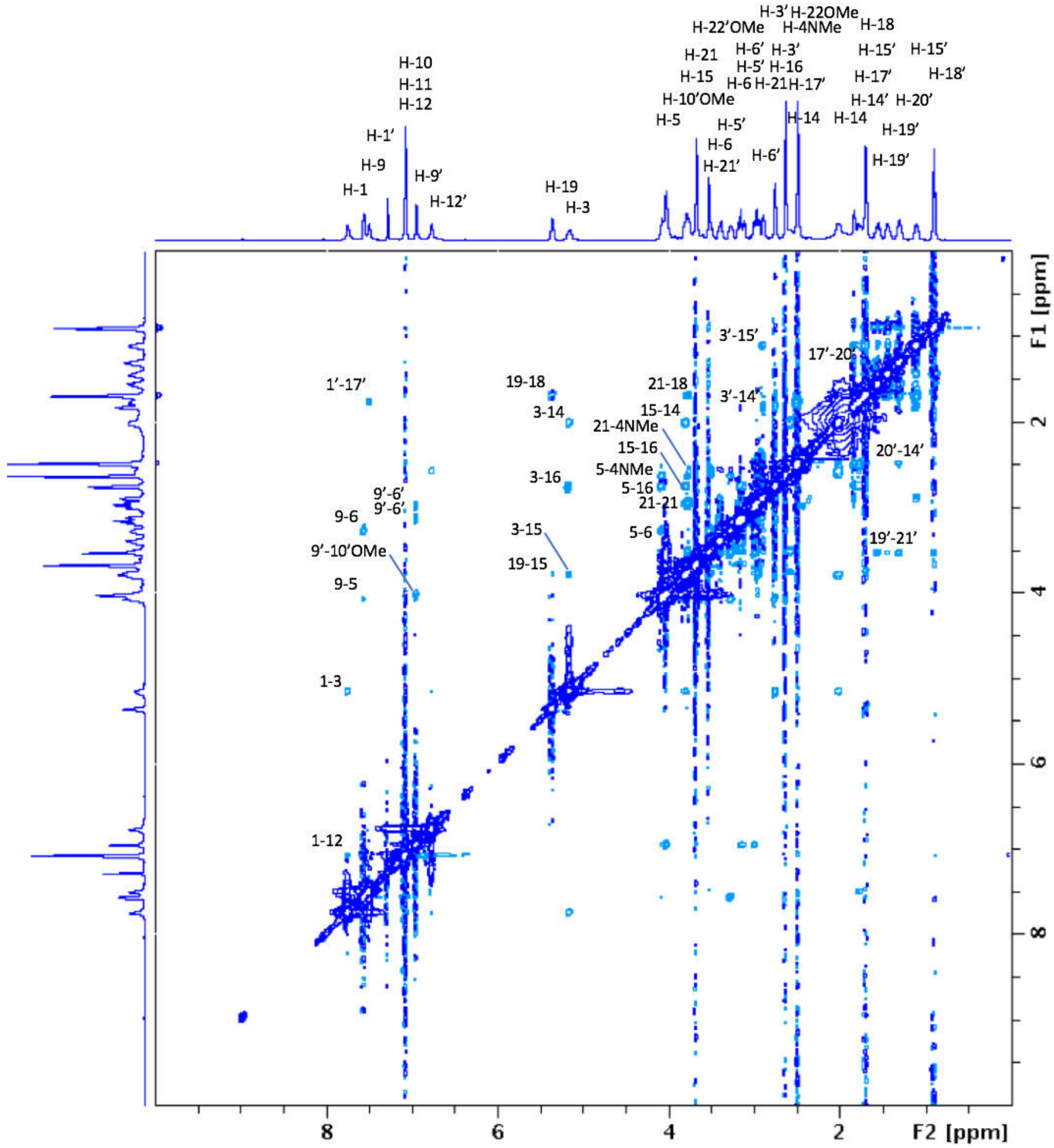

Figure S6: ^{1}H-^{1}H NOESY spectrum of voacamine A. Atom labels on the 1D spectrum are positioned so the centre of the H corresponds to the resonant frequency.

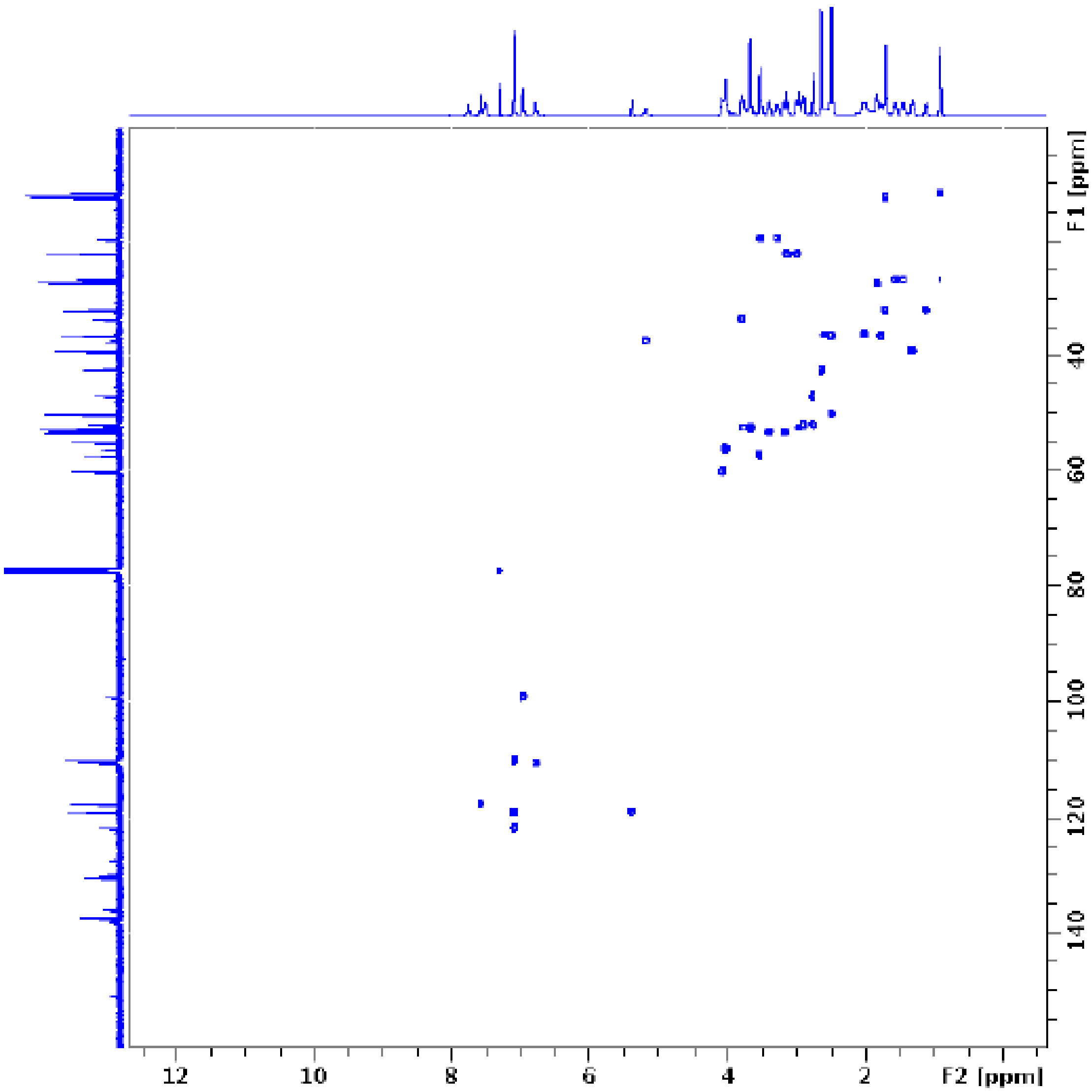

Figure S7: ^{1}H-^{13}CHSQC spectrum of voacamine A.

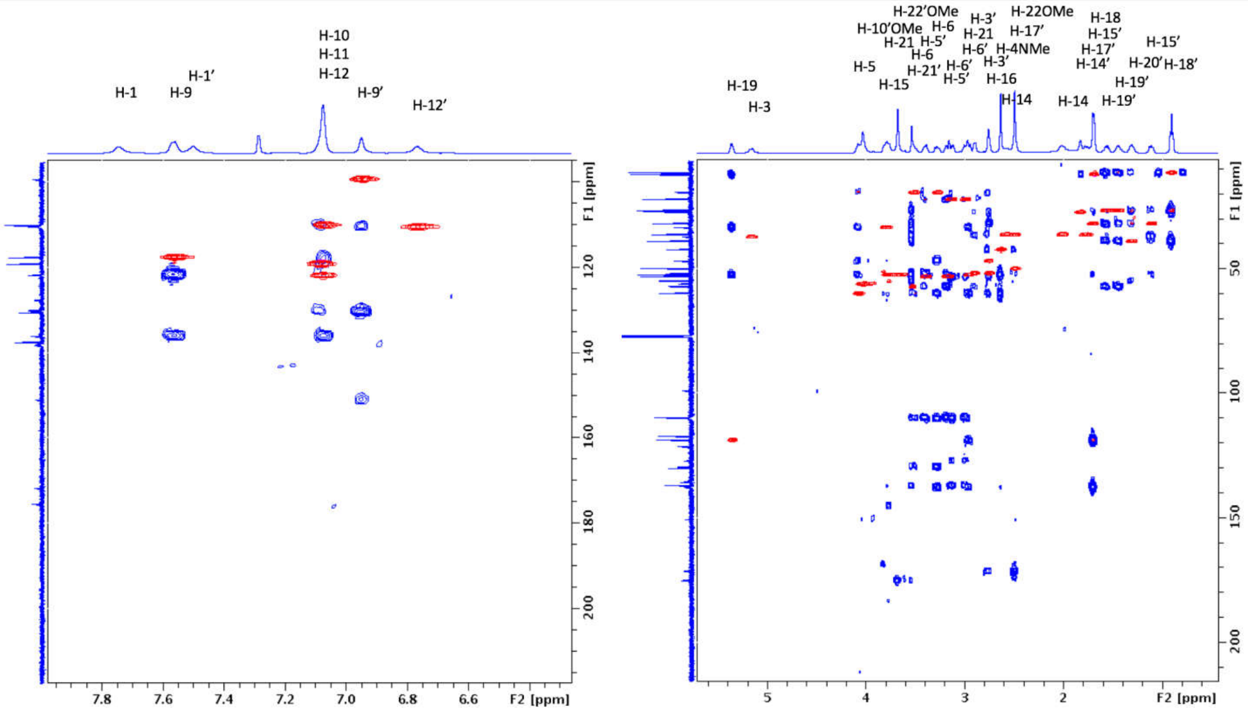

Figure S8: ^{1}H-^{13}CHSQC and ^{1}H-^{13}C HMBC spectra of voacamine A. Atom labels on the 1D ^{1}H spectrum are positioned so the centre of the H corresponds to the resonant frequency.

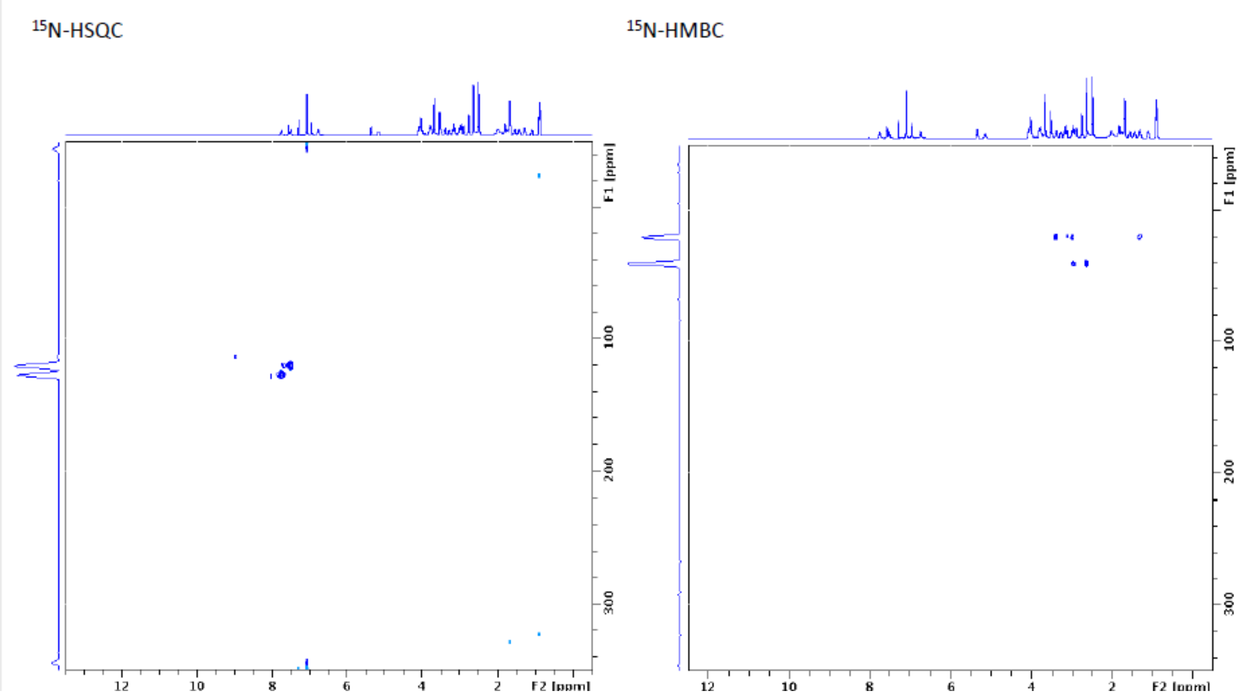

Figure S9: ^{1}H-^{15}N-HSQC and ^{1}H-^{15}N-HMBC spectra of voacamine A.

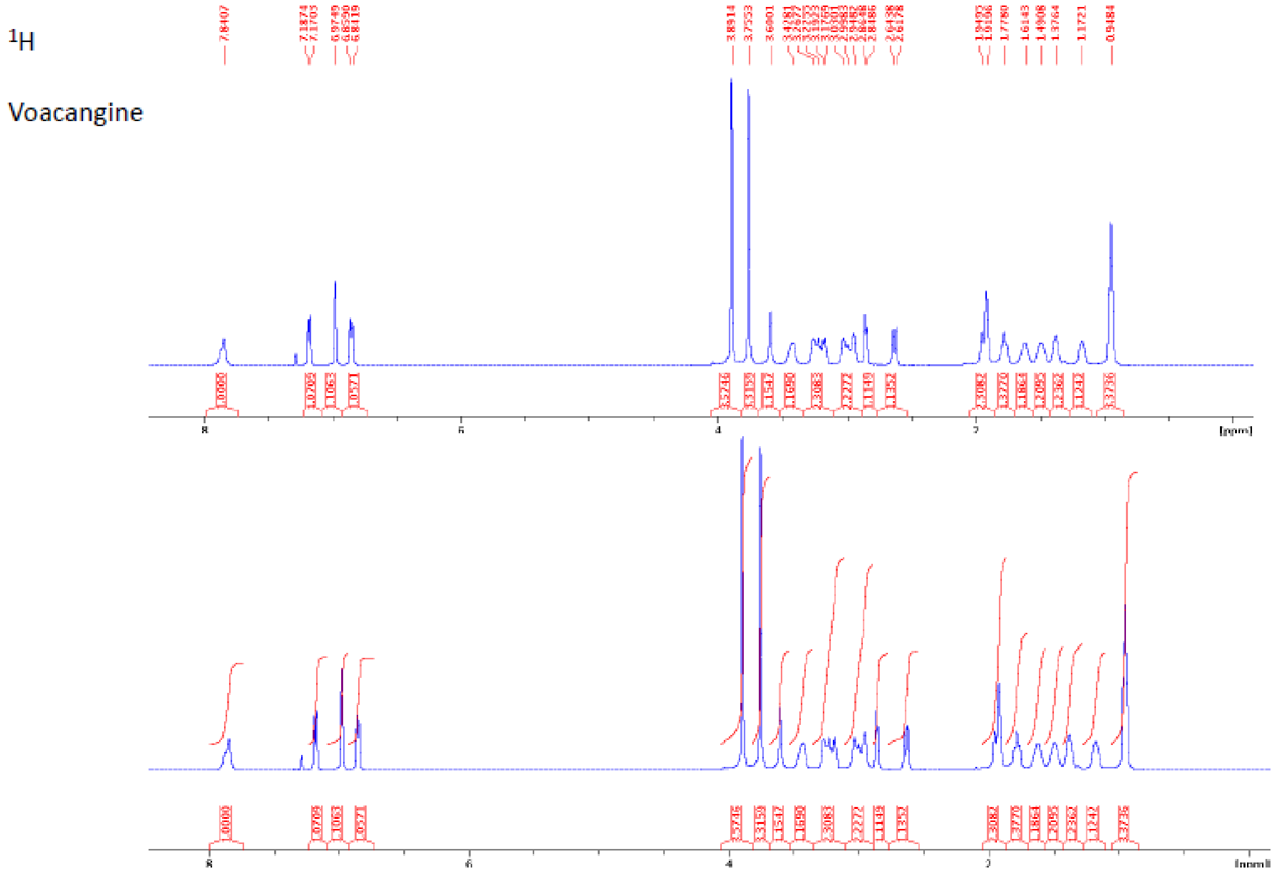

Figure S10: ¹H NMR (500 MHz, $CDCl_3$) spectrum of voacangine.

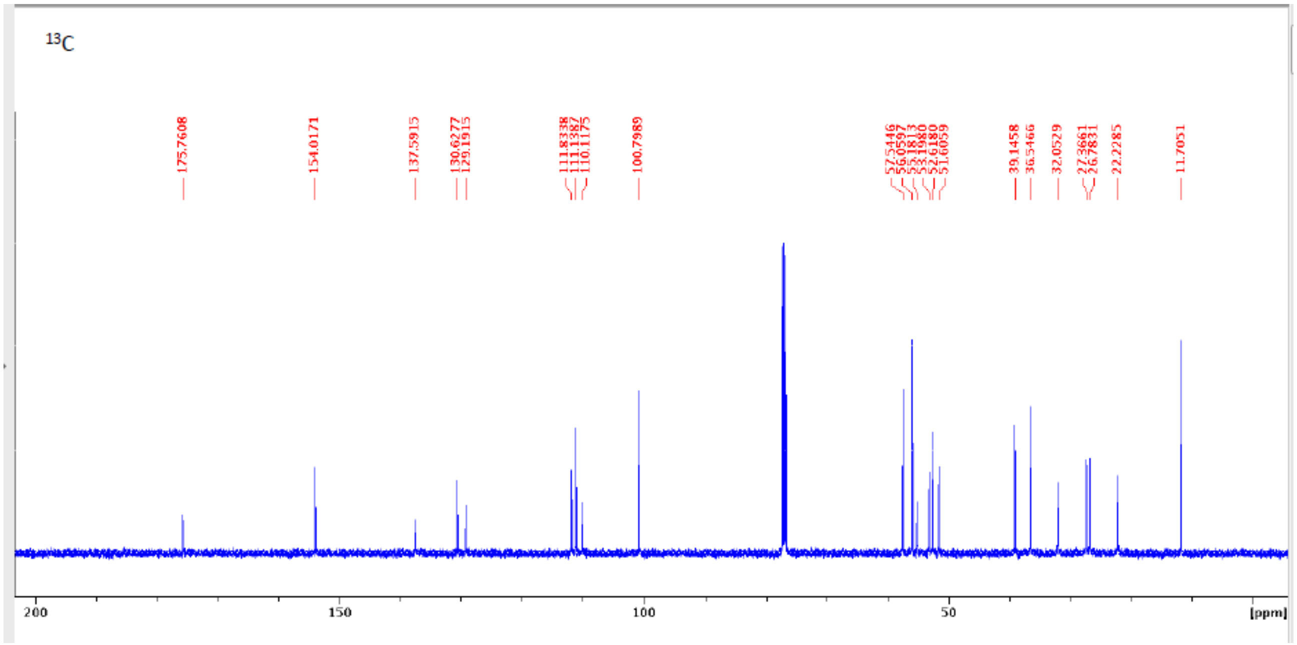

Figure S11: ^{13}C NMR (125 MHz, $CDCl_3$) spectrum of voacangine.

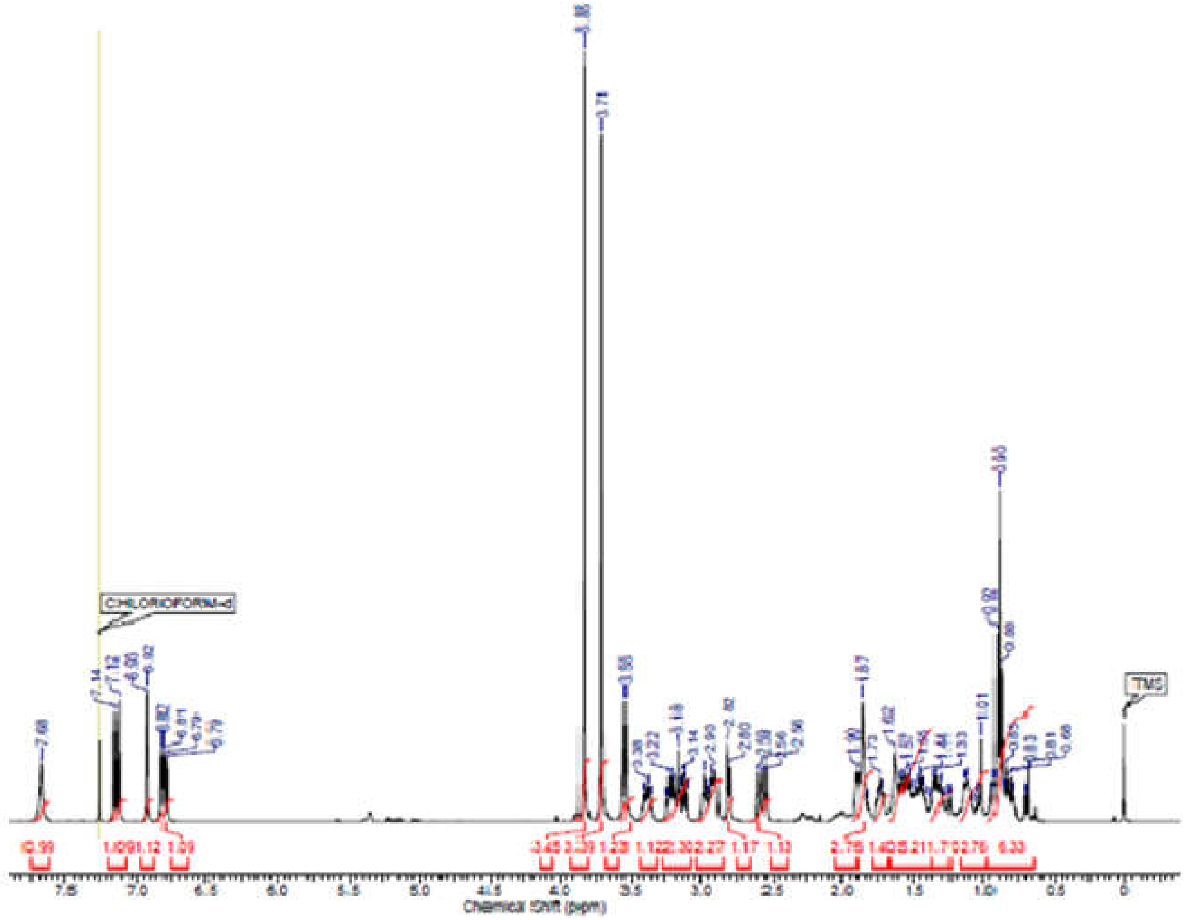

Figure S12: ^{1}H NMR (500 MHz, $CDCl_3$) spectrum of voacristine.

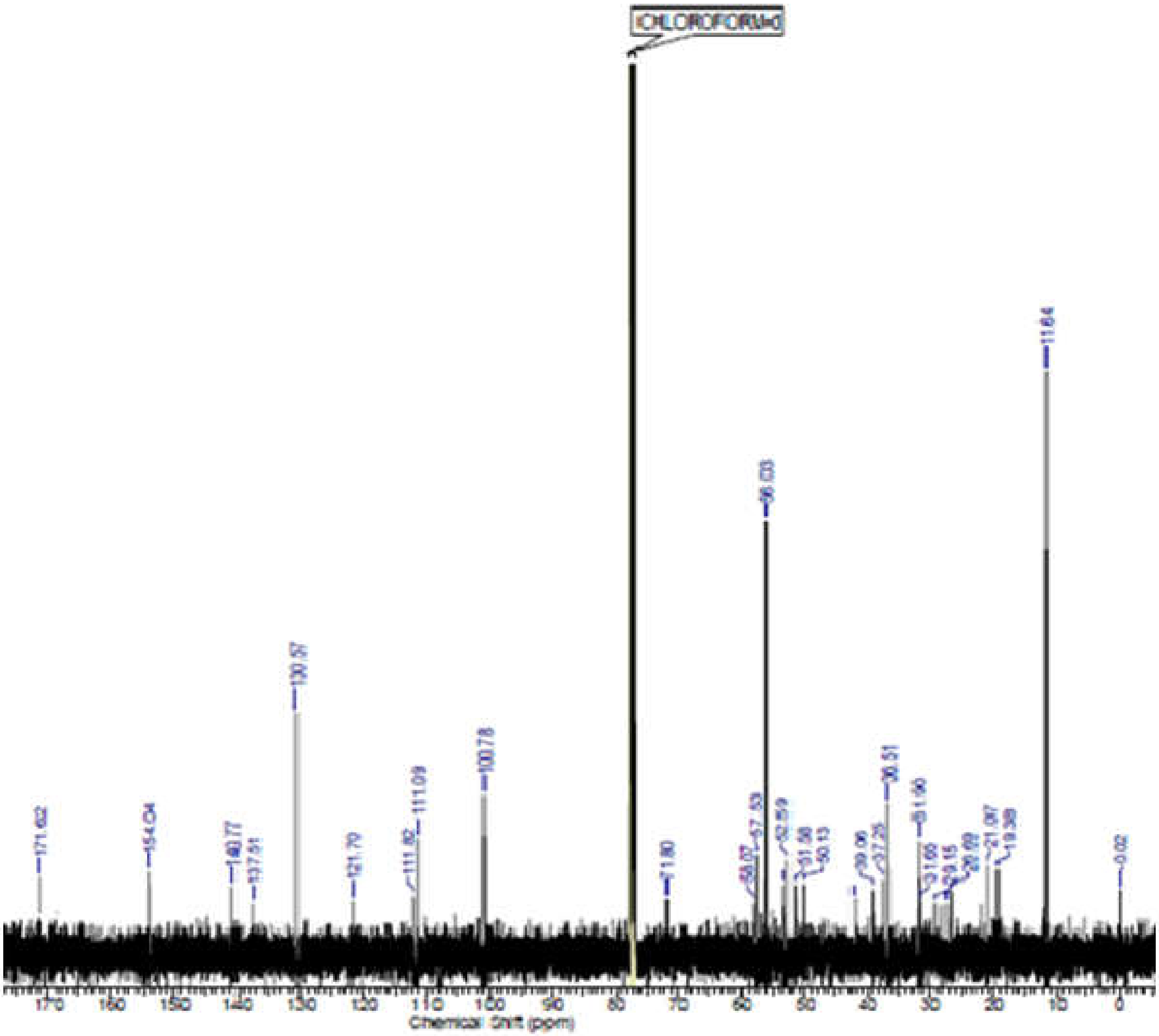

Figure S13: ^{13}C NMR (125 MHz, $CDCl_3$) spectrum of voacristine.

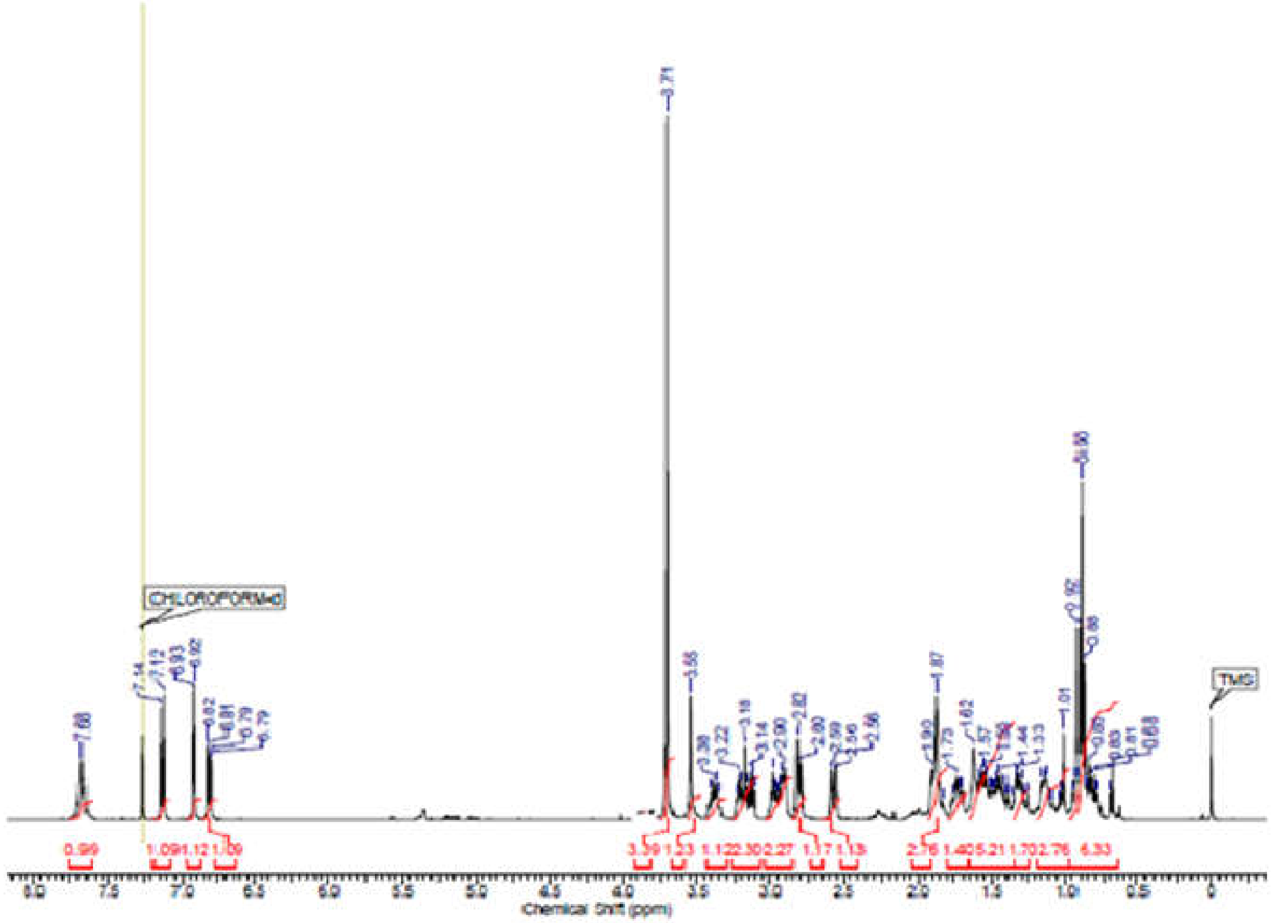

Figure S14: ^{1}H NMR (500 MHz, $CDCl_3$) spectrum of coronaridine.

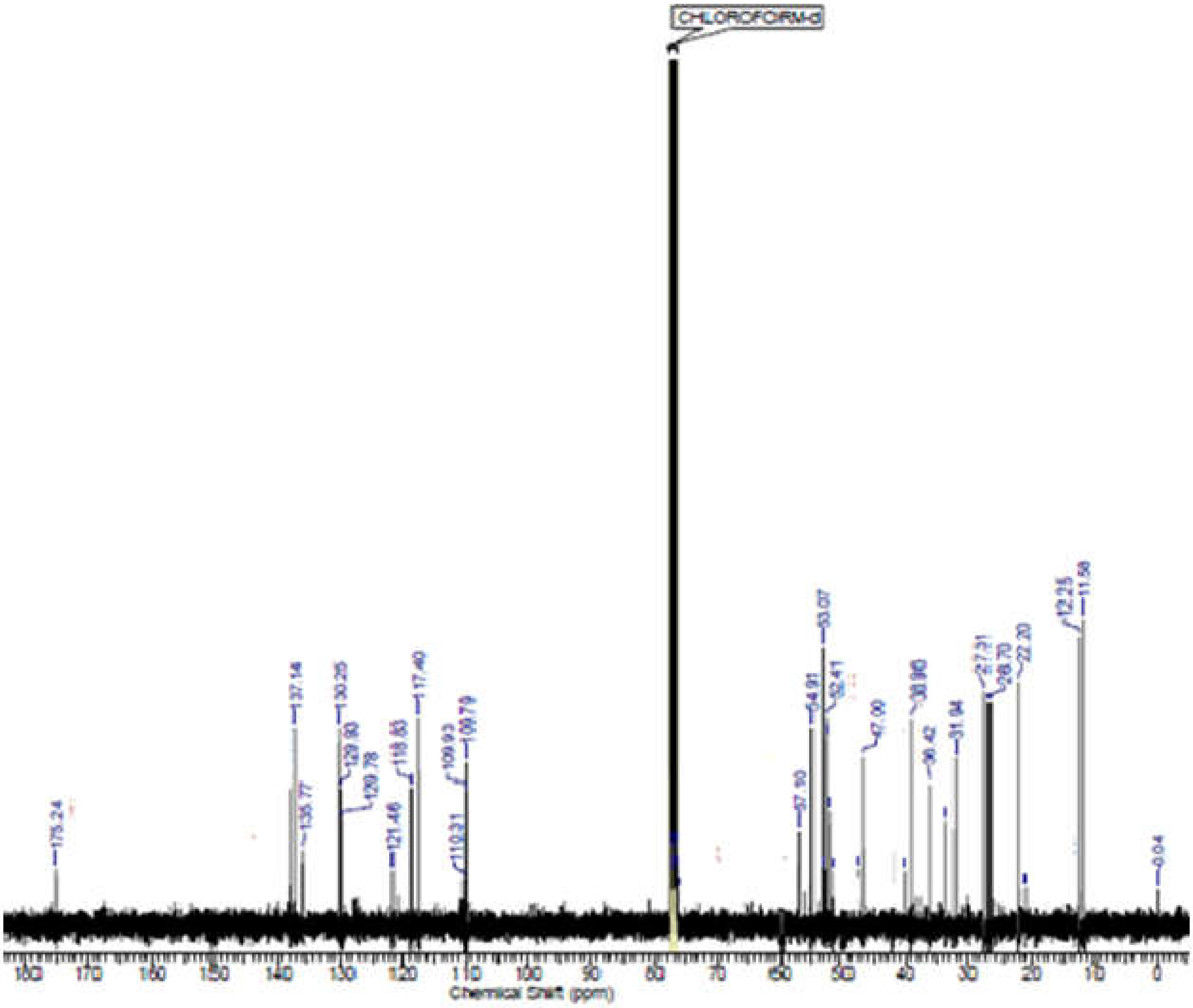

Figure S15: ^{13}C NMR (125 MHz, $CDCl_3$) spectrum of coronaridine.

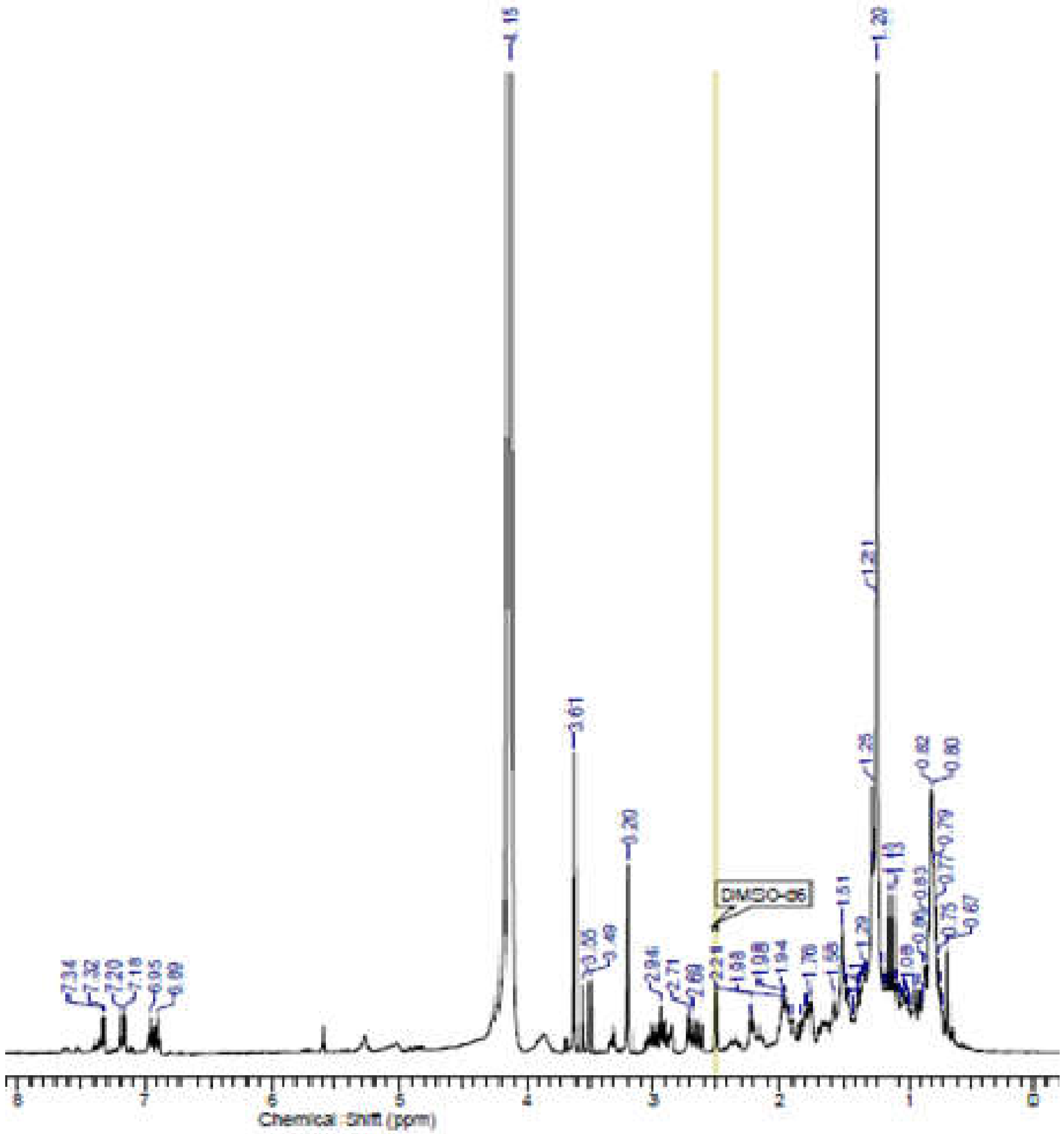

Figure S16: ^{1}H NMR (500 MHz, DMSO-d_6) spectrum of tabernanthine.

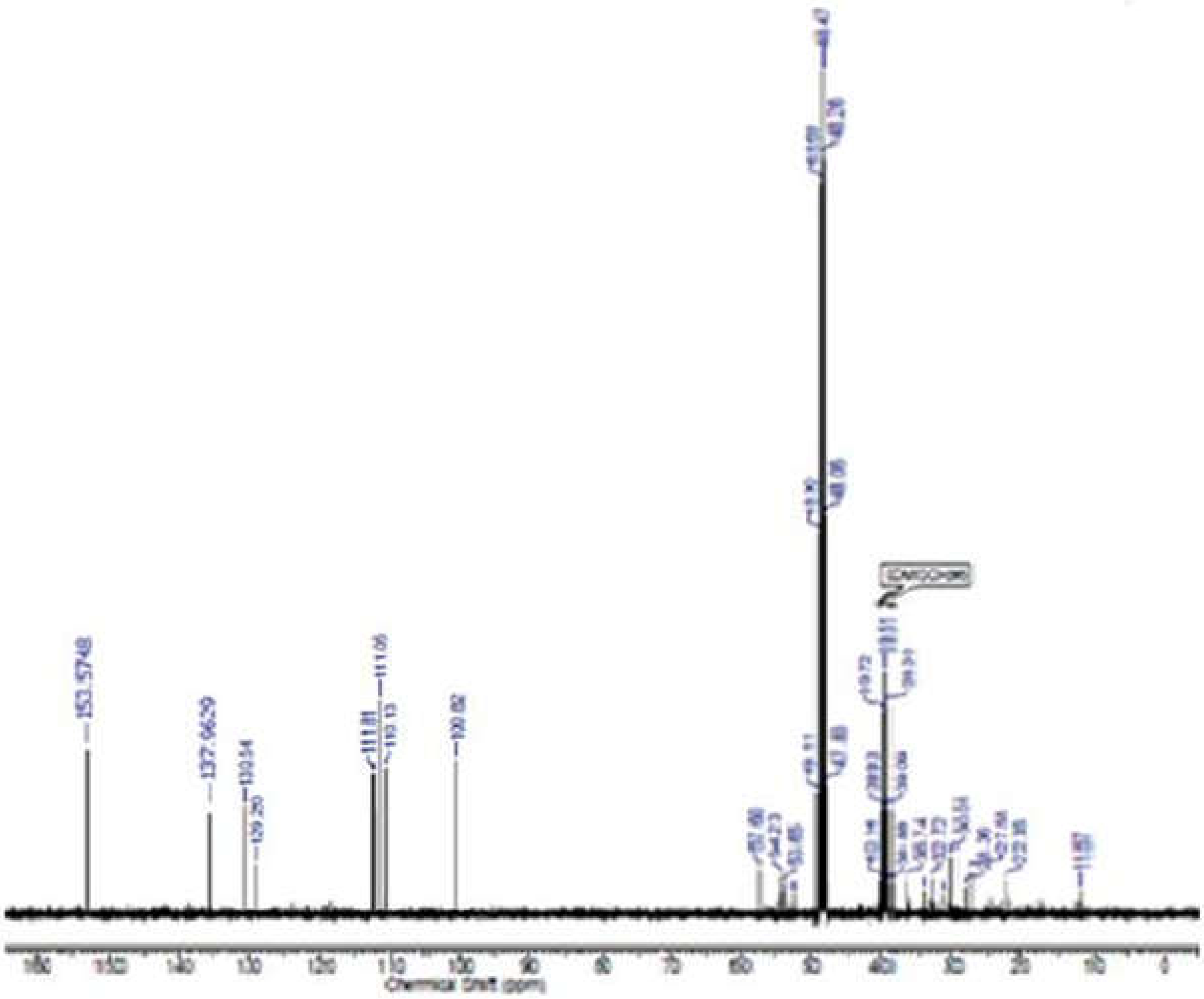

Figure S17: [13] C NMR (125 MHz, MeOD) spectrum of tabernanthine.

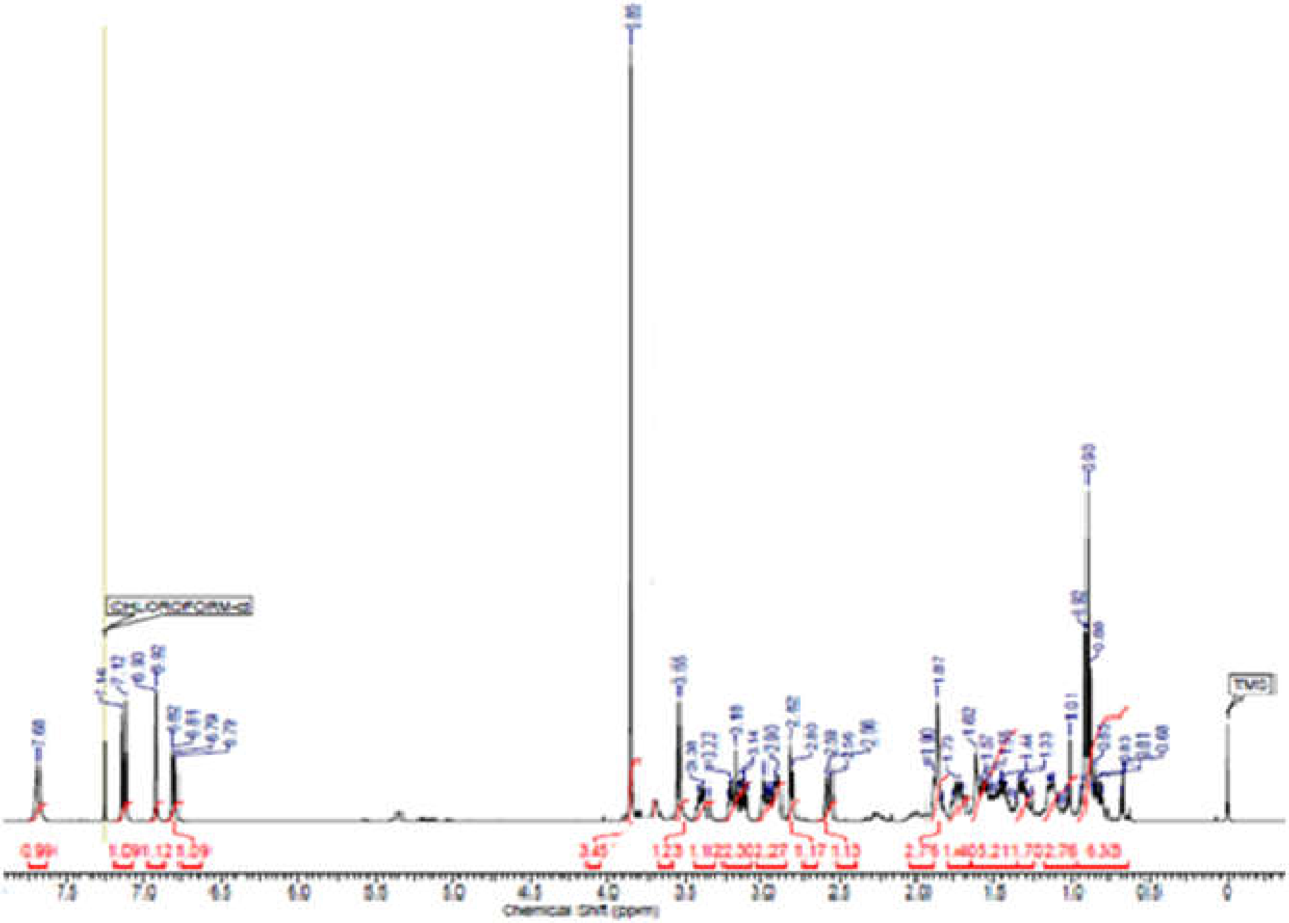

Figure S18: ^{1}H NMR (500 MHz, $CDCl_3$) spectrum of iboxygaine.

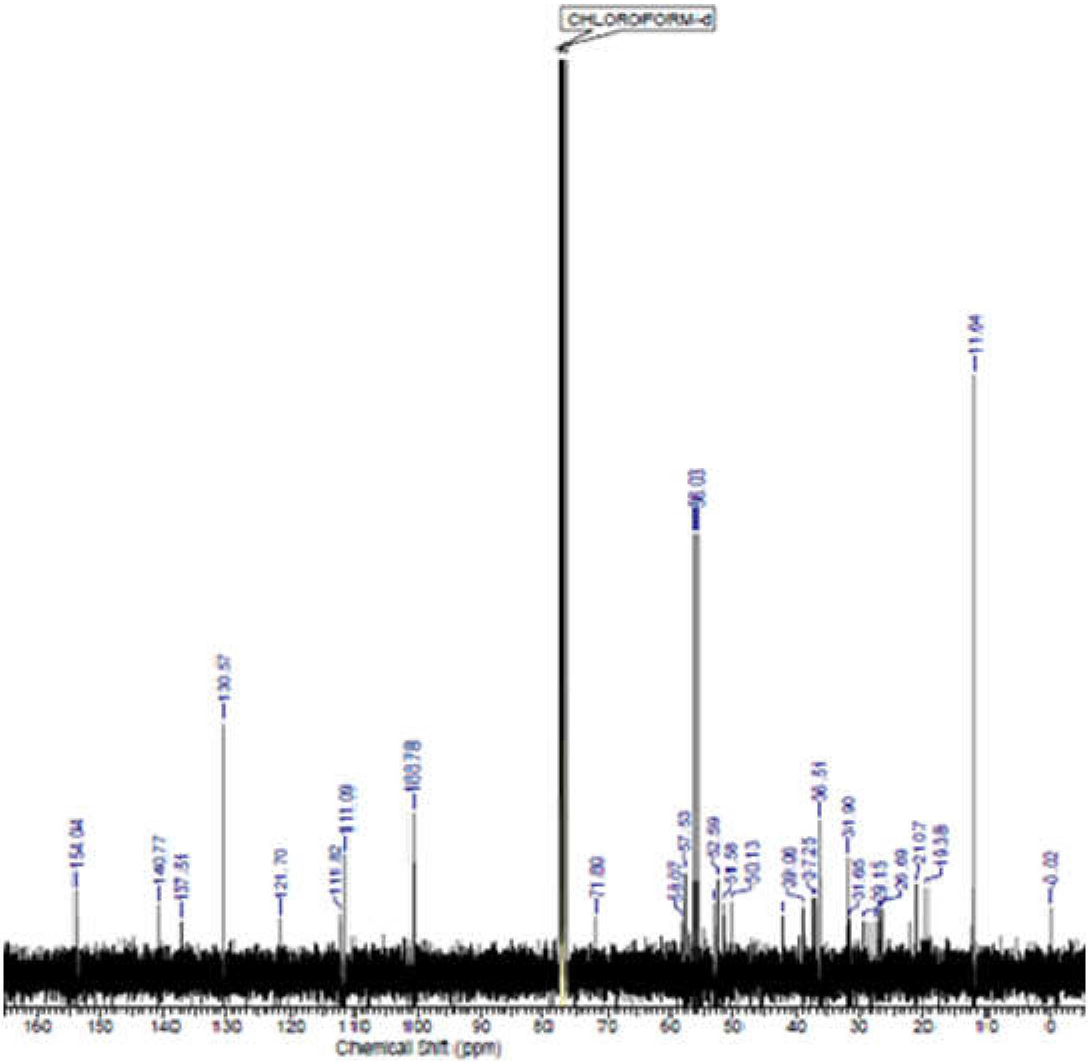

Figure S19: ^{13}C NMR (125 MHz, $CDCl_3$) spectrum of iboxygaine.

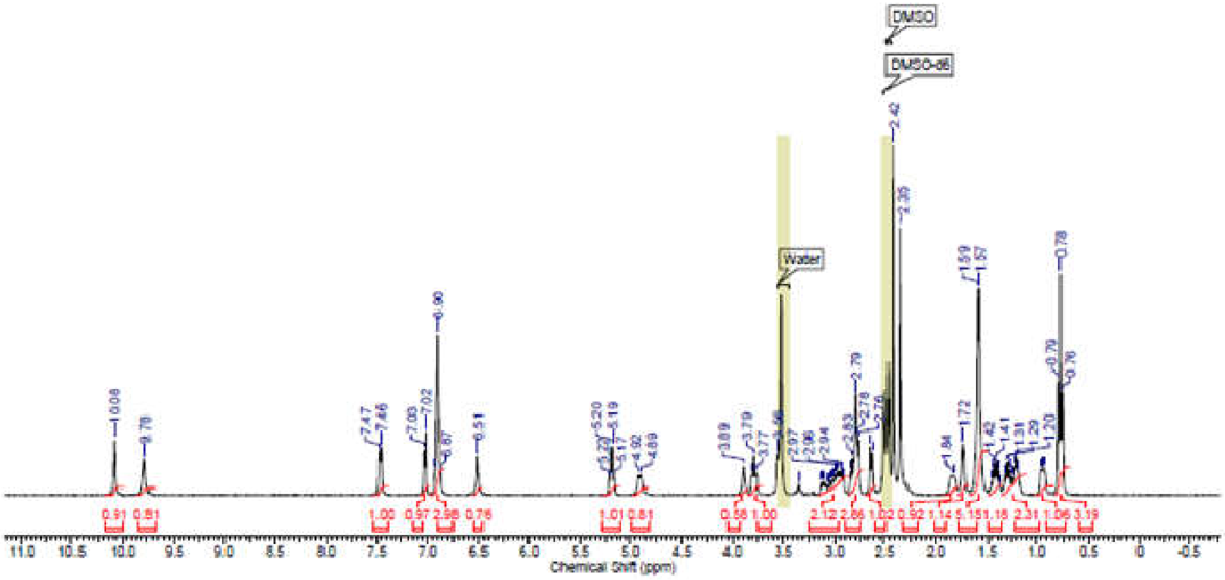

Figure S20: ^{1}H NMR (500 MHz, DMSO-d_6) spectrum of voacamine.

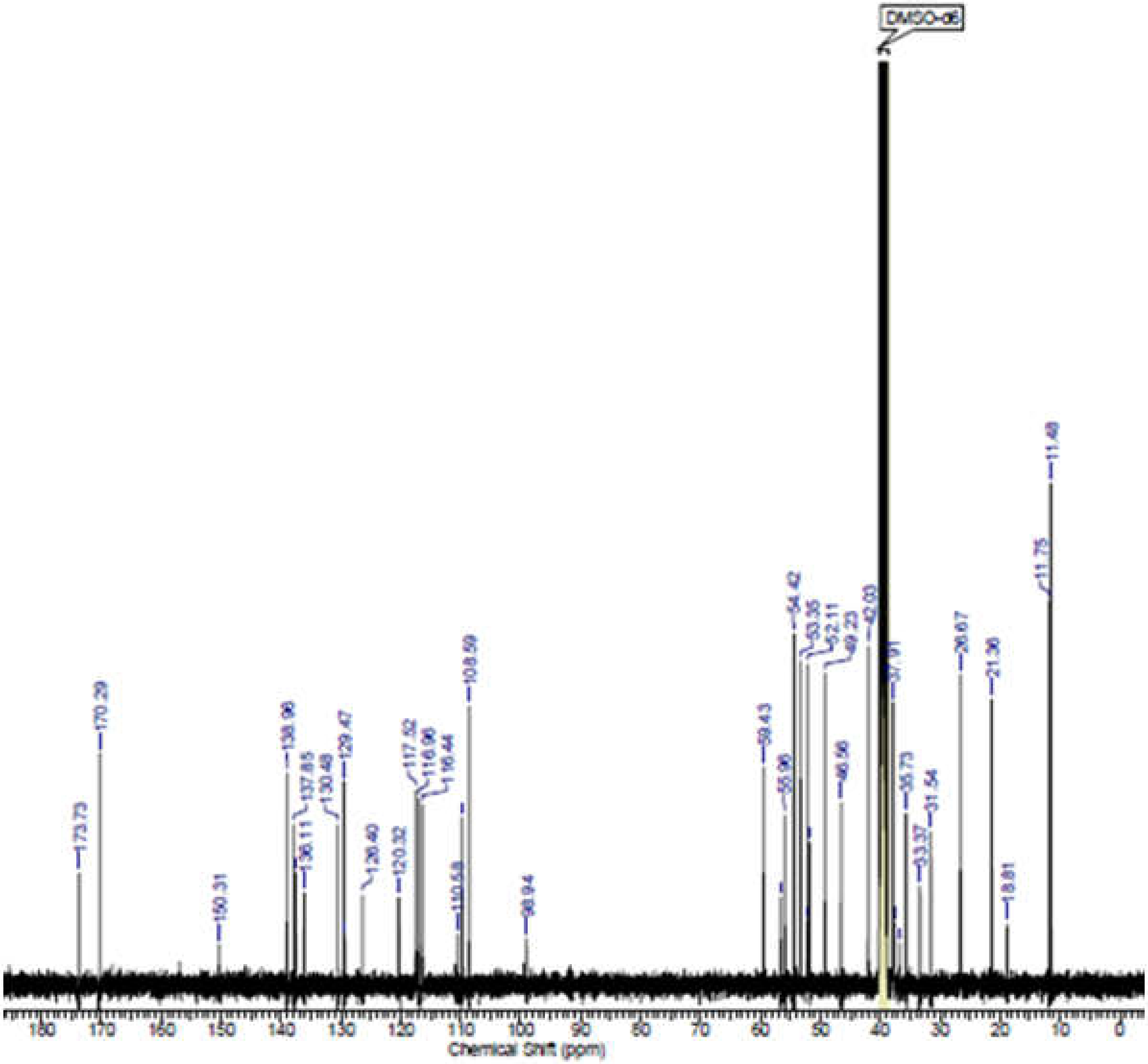

Figure S21: ^{13}C NMR (125 MHz, DMSO-d_6) spectrum of voacamine.

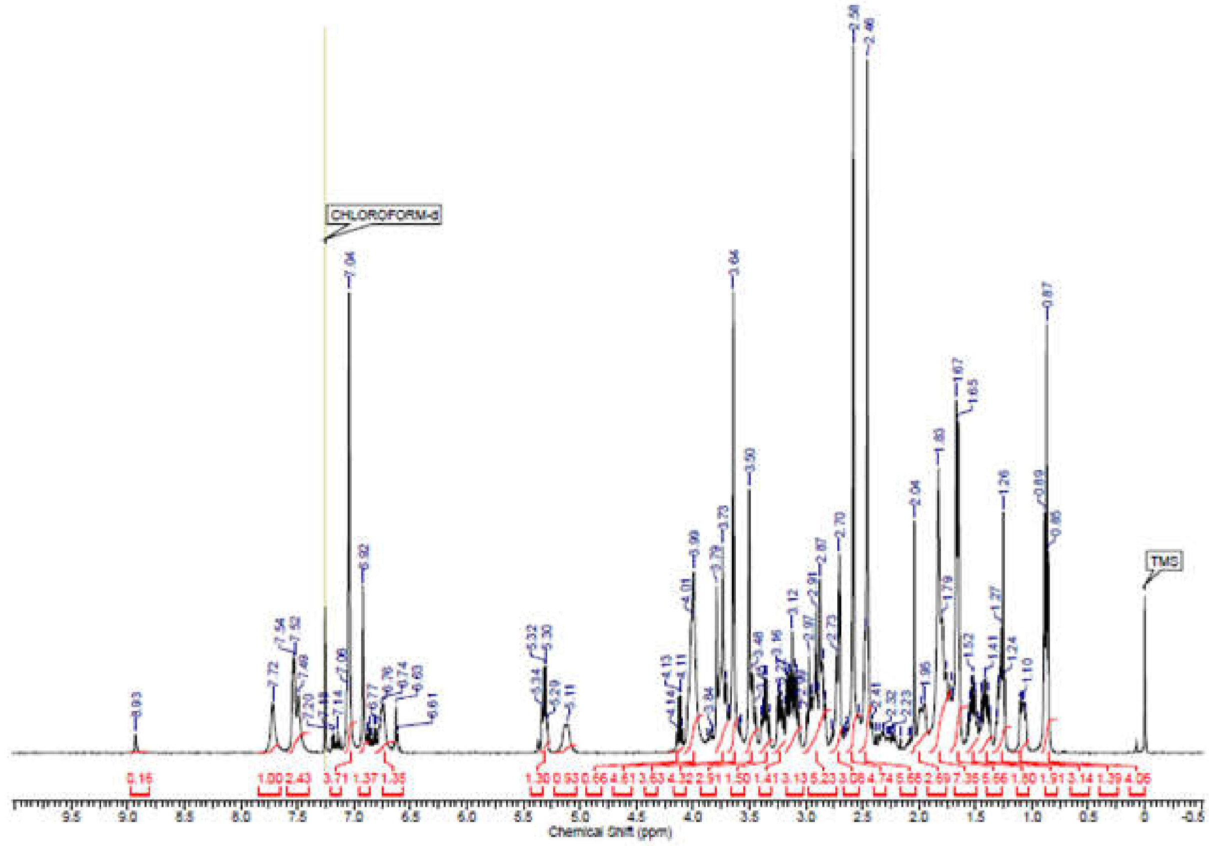

Figure S22: ^{1}H NMR (500 MHz, $CDCl_3$) spectrum of voacorine.

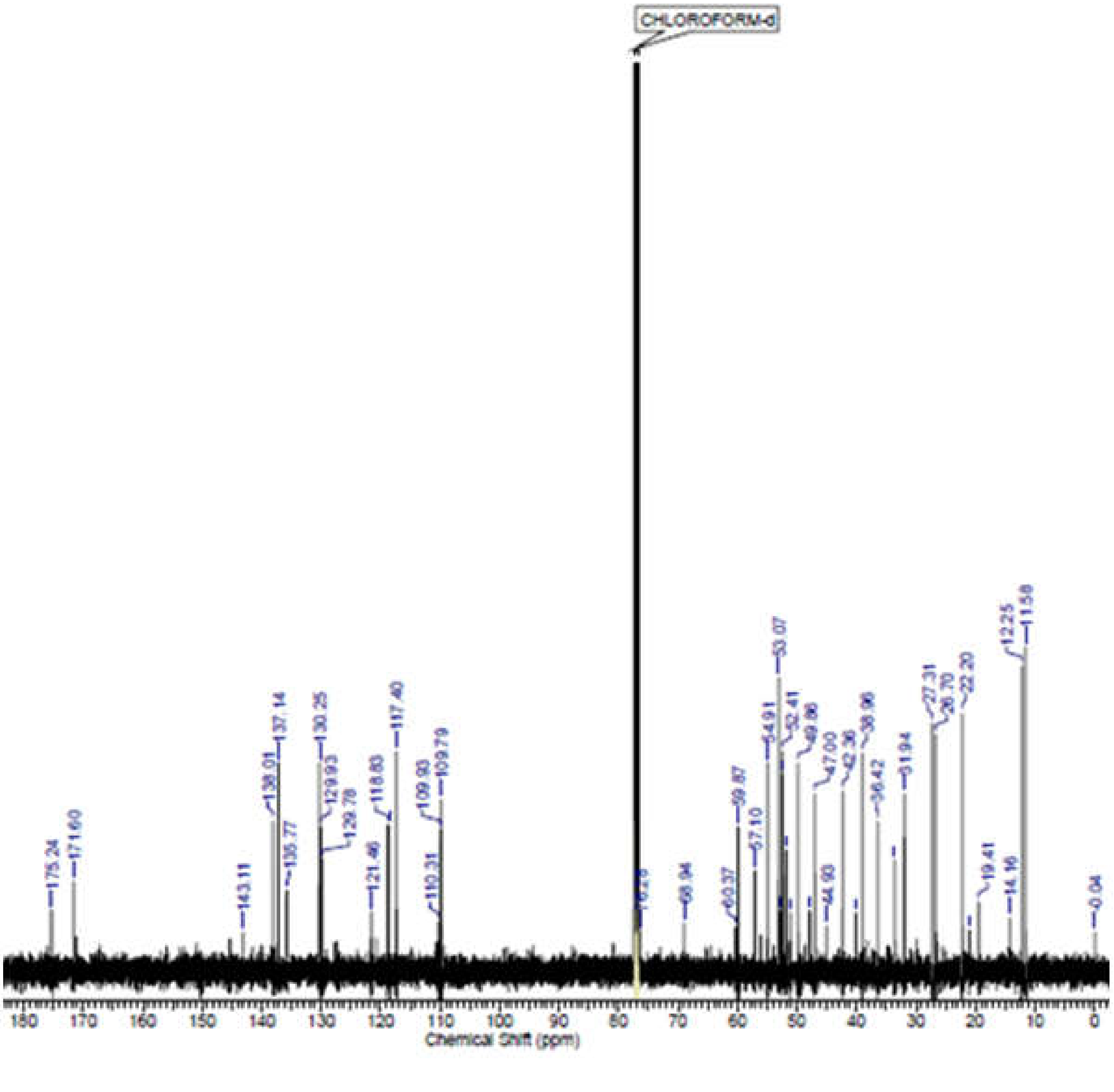

Figure S23: ^{13}C NMR (125 MHz, $CDCl_3$) spectrum of voacorine.

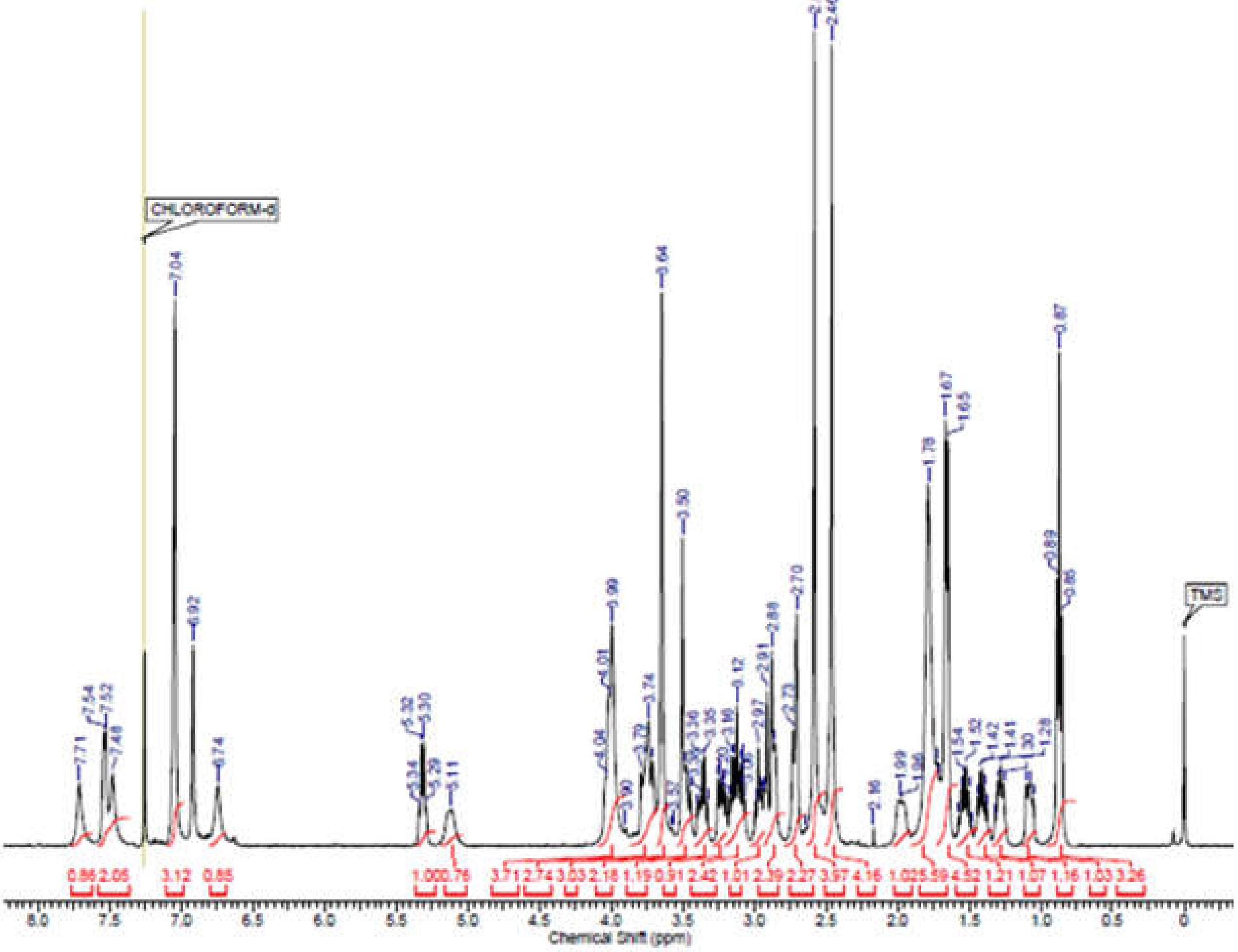

Figure S24: ^{1}H NMR (500 MHz, $CDCl_3$) spectrum of conoduramine.

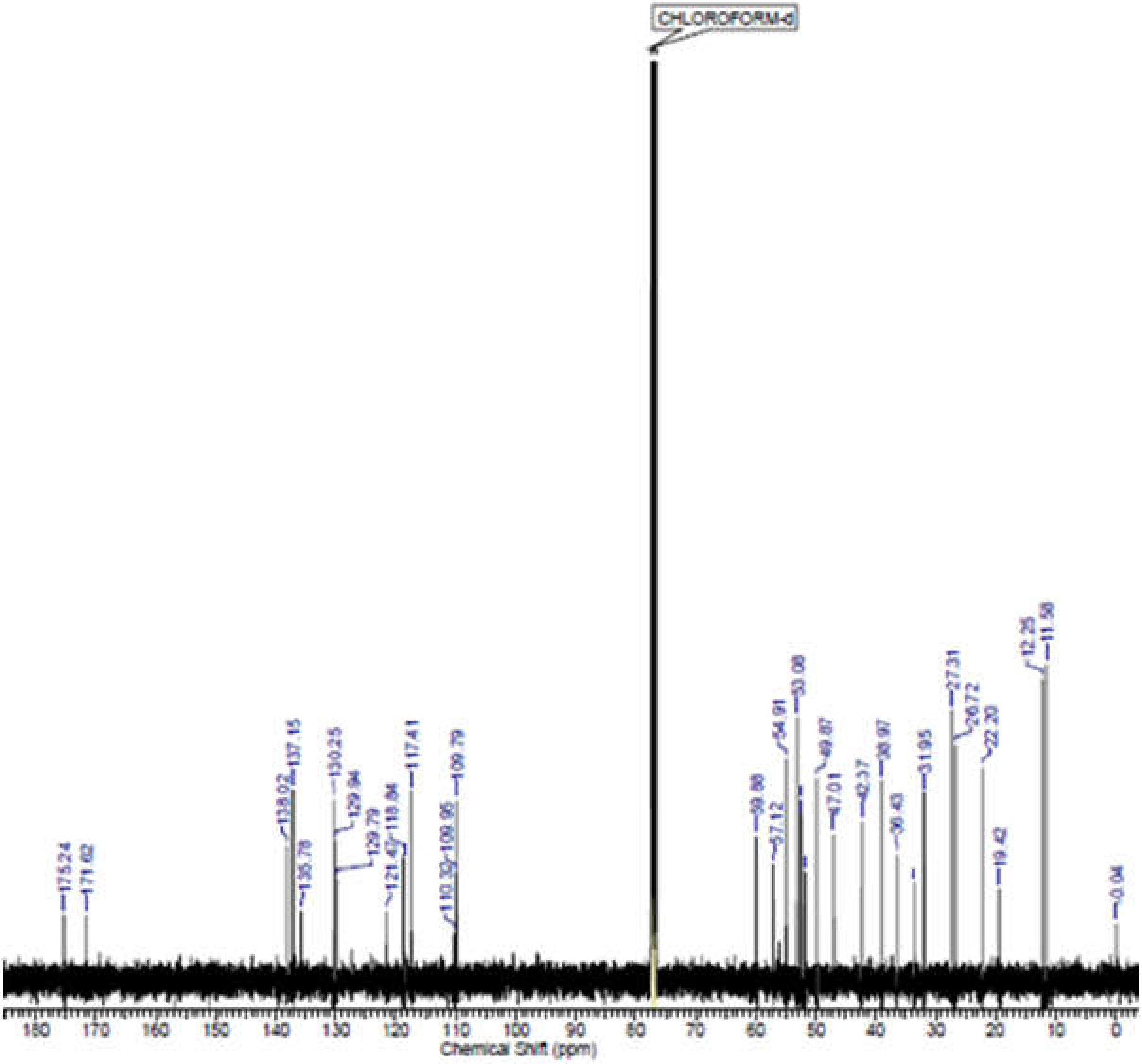

Figure S25: [13] C NMR (125 MHz, $CDCl_3$) spectrum of conoduramine.

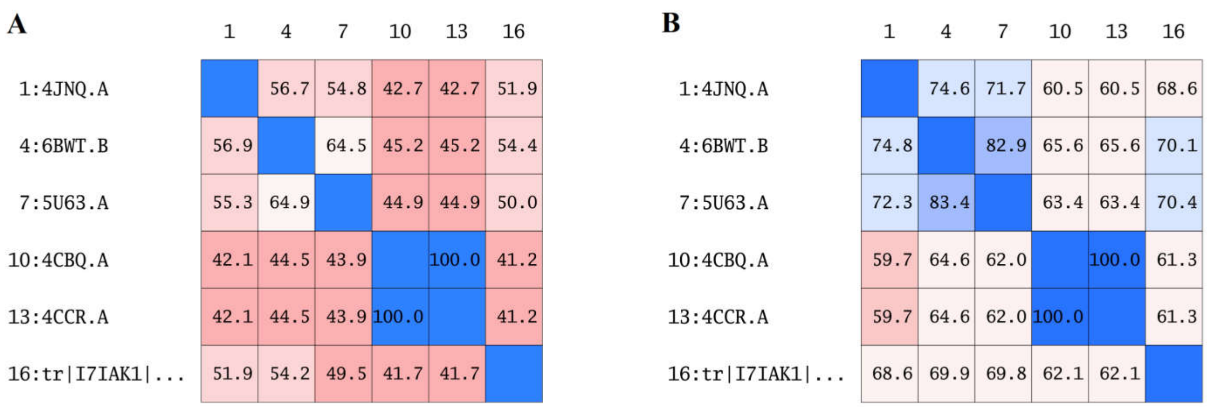

Figure S26:Percentage sequence identity and similarity values to our target.

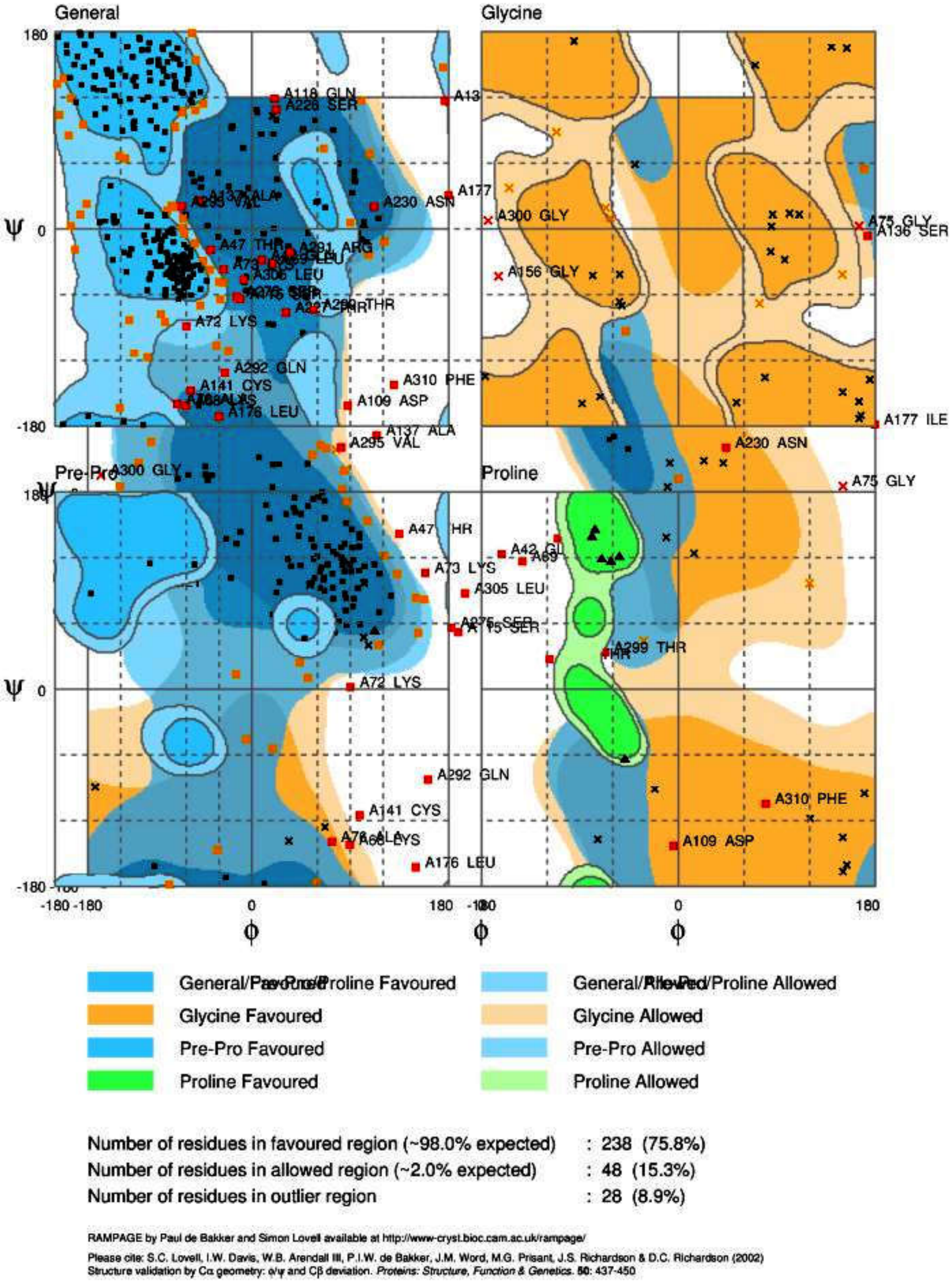

Figure S27: Ramachandran plot (φ/ψ) distribution of the backbone conformation.

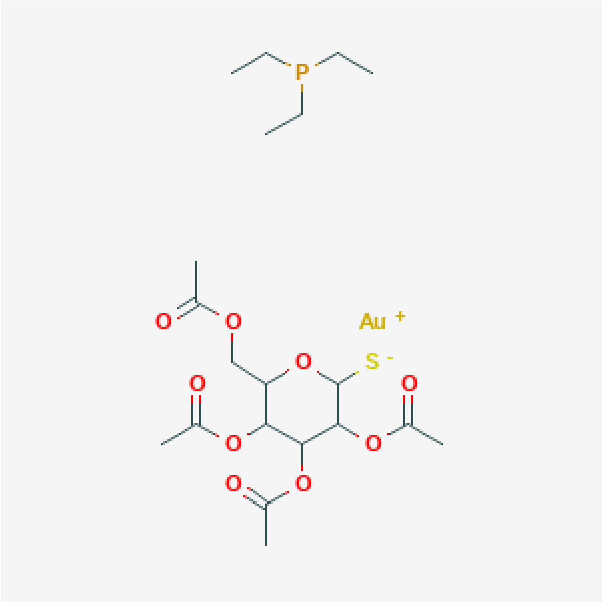

Figure S28: Chemical structure of auranofin.

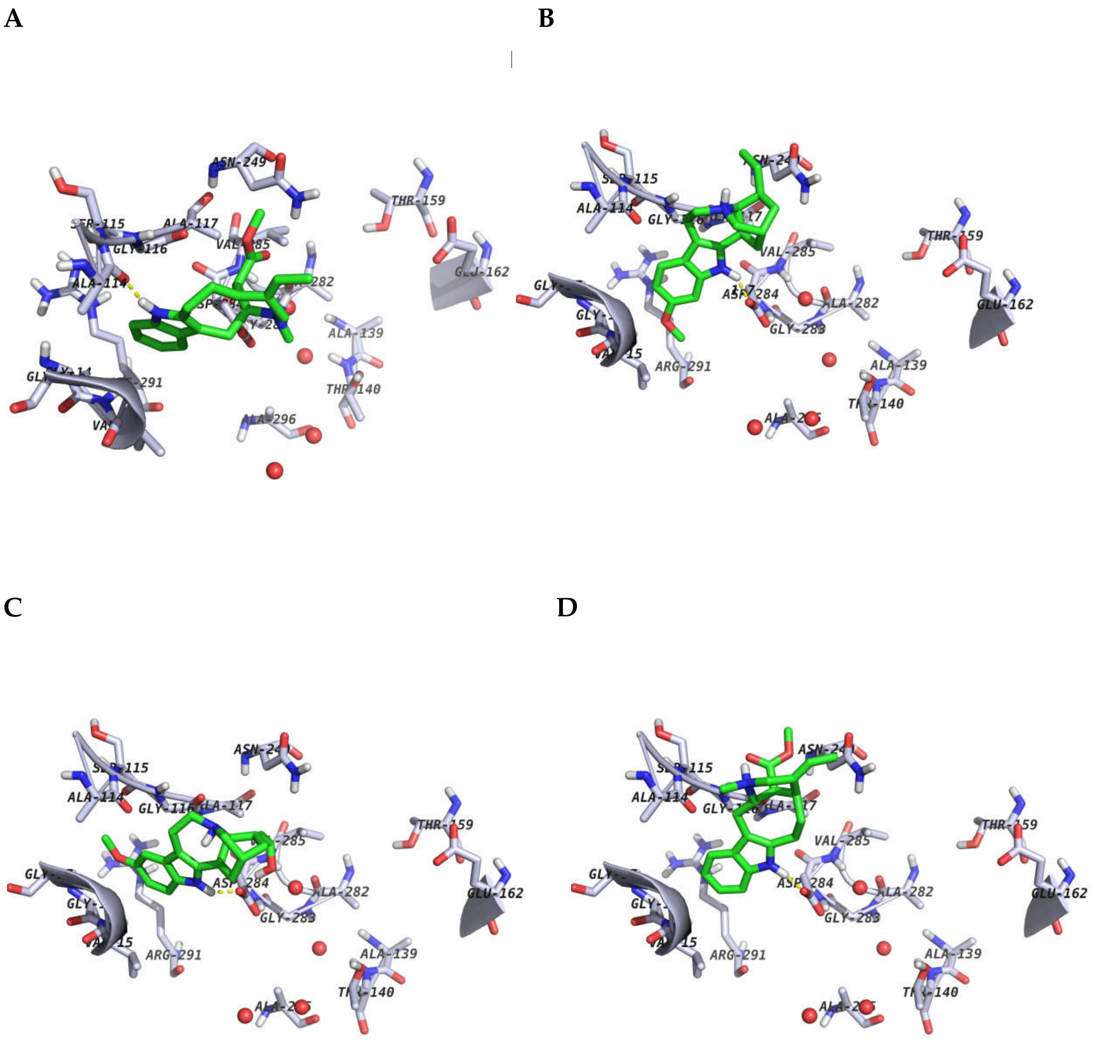

Figure S29: Docking poses of (A) compound **1a**, (B) compound **5**, (C) compound **6**, (D) compound **7a**.

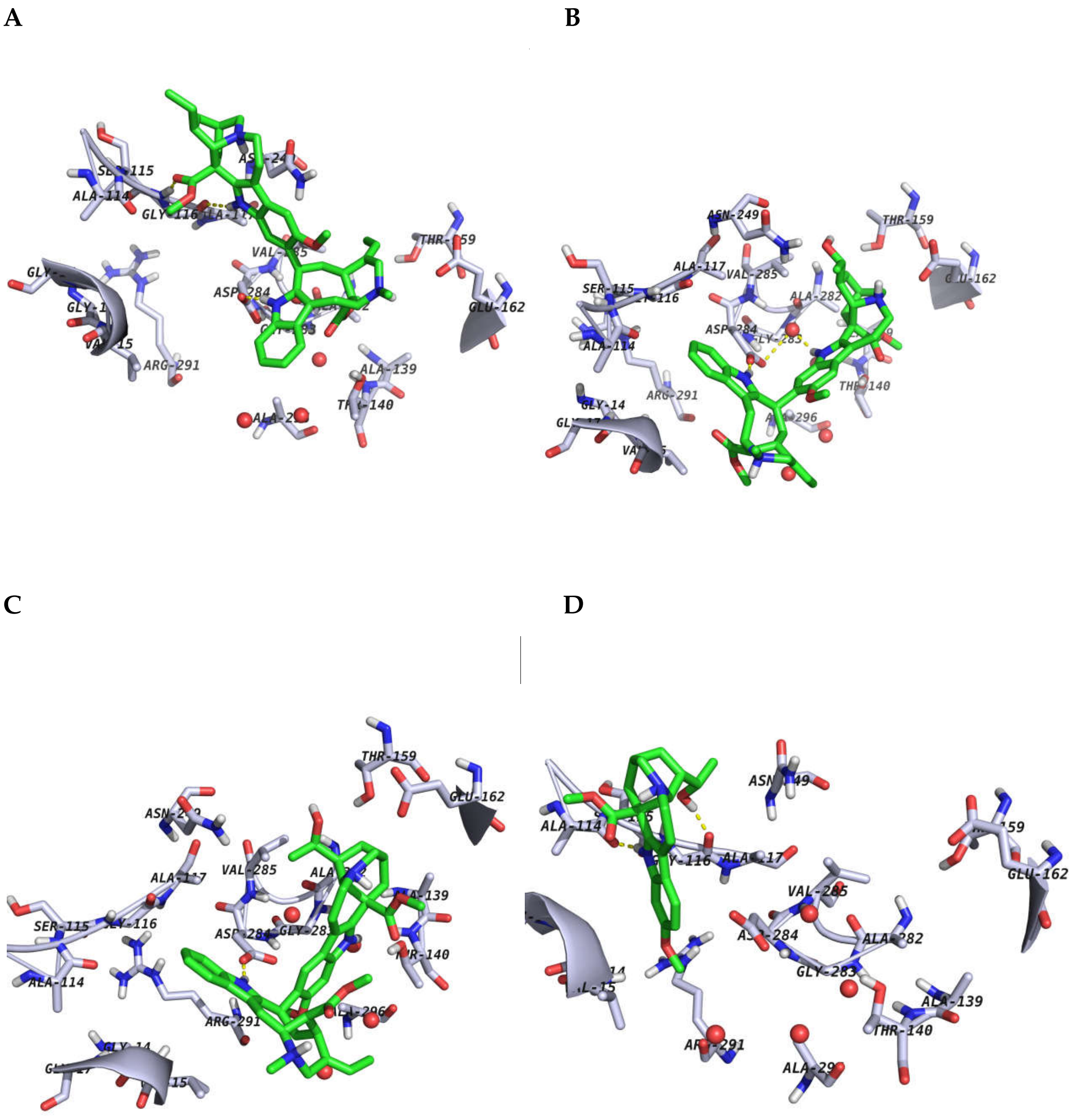

Figure S30: Docking poses of (A) compound **7**, (B) compound **8**, (C) compound **9**, (D) compound **9b**.
